# Evaluation of perinatal autonomic development in infants using the QT/RR variability ratio

**DOI:** 10.20407/fmj.2019-006

**Published:** 2019-11-02

**Authors:** Yuri Mizutani, Arisa Kojima, Yuka Takeuchi, Hirofumi Kusuki, Keiko Sugimoto, Keisuke Osakabe, Naohiro Ichino, Masayuki Fujino, Kazuyoshi Saito, Masafumi Miyata, Tsuneaki Sadanaga, Tadayoshi Hata

**Affiliations:** 1 Graduate School of Health Sciences, Fujita Health University, Toyoake, Aichi, Japan; 2 Department of Joint Research Laboratory of Clinical Medicine, Fujita Health University Hospital, Toyoake, Aichi, Japan; 3 Department of Pediatrics, Fujita Health University, School of Medicine, Toyoake, Aichi, Japan; 4 Seigato Hospital, Kumamoto, Kumamoto, Japan

**Keywords:** Gestational age, Myocardial repolarization, Variability ratio, Heart rate variability

## Abstract

**Objectives:**

Development of the autonomic nervous system may play a role in myocardial repolarization lability in infants, but its relationship to repolarization abnormalities remains unclear. Thus, the aim of the present study was to evaluate the relationship between gestational age and ventricular repolarization lability using the variability ratio (VR).

**Methods:**

Infants who underwent electrocardiography at a 1-month check-up were included (n=209; 125 males). Gestational age and the following four VR parameters at 1 month of age were compared: VR-I, SDQT/SDRR; VR-II, SDQT/rMSSD; VR-III, SDQTc/SDRR; and VR-IV, SDQTc/rMSSD; where SD, QTc, and rMSSD are standard deviation, QT interval corrected using Fridericia’s formula, and root mean square difference of successive RR intervals, respectively. Twenty-eight preterm infants born at <37 weeks of gestation and 181 full-term infants were included.

**Results:**

Significant correlations were observed between gestational age and VR-I, -III, and -IV (all p<0.05). All VR values were significantly higher in preterm infants compared with full-term infants (I: 0.54 vs 0.48, II: 1.15 vs 0.96, III: 0.88 vs 0.68, IV: 1.59 vs 1.39; median, all p<0.05).

**Conclusion:**

VR assessed at 1 month after birth was impaired in preterm infants, suggesting immaturity of their cardiac autonomic nervous system and ventricular myocardial repolarization.

## Introduction

Japan is currently appreciated as the “nation with the world’s lowest neonatal mortality rate”.^1,2^ However, there were still 96 deaths due to sudden infant death syndrome (SIDS) in Japan in 2015, making SIDS the third leading cause of infant death.^3^ SIDS often occurs at 1–4 months of age, with a higher incidence among preterm infants than full-term infants. Although the pathology of SIDS is not fully understood, dysfunction of autonomic nervous control of the respiratory and cardiovascular systems might contribute.^4^

Heart rate variability (HRV), a parameter of autonomic nervous control of the heart, involves variations in RR interval.^5–7^ Notably, reduced frequency power was observed for the HRV of infants who suddenly died because of SIDS.^8–10^ The QT interval reflects the action potential duration of the ventricular myocardium. Instability in the repolarization process (i.e., reduced beat-to-beat pattern) can make the substrate susceptible to fatal arrhythmia.^11^ However, few studies have examined temporal variations in the repolarization process among infants, or their development of autonomic nervous function.^8,9^ In adults, QT interval variability has been extensively investigated to identify abnormalities in the repolarization process.^11^ In these studies, the variability ratio (VR, defined as the ratio between the standard deviation of all QT intervals and standard deviation of all RR intervals) was calculated from a single lead. VR can also be applied in infants if electrocardiogram (ECG) recordings have a stable baseline.^12,13^

We previously reported physiological changes in ventricular repolarization variability using body surface ECG, and assessed developmental changes in the autonomic nervous system of infants and school-aged children.^14,15^ However, changes in the autonomic nervous system related to the ventricular myocardium, which can be evaluated by assessing the QT interval, have not been reported for preterm infants.

In the present study, we hypothesized that VR in 1-month-old infants is affected by their gestational age. Thus, we investigated the association between myocardial repolarization lability index, namely the VR, and the perinatal profile of healthy 1-month-old infants.

## Methods

### Participants

The sample population comprised 230 infants who were delivered and underwent a 1-month postnatal checkup at Fujita Health University from October 2014 to November 2015. One infant with patent ductus arteriosus was excluded, as were 20 full-term infants with rapid heart rates exceeding 180 beats per minute because the P wave overlapped the end of the preceding T wave, which made their record unsuitable for automated analysis. Accordingly, 209 infants were included in our analysis. All procedures involving human participants were performed in accordance with institutional ethical standards (No. 14-188) and/or National Research Committee, and with the 1964 Helsinki declaration and its later amendments or comparable ethical standards. Informed consent was obtained in writing from each child’s parents/guardians.

### Procedures

**Figure 1:**
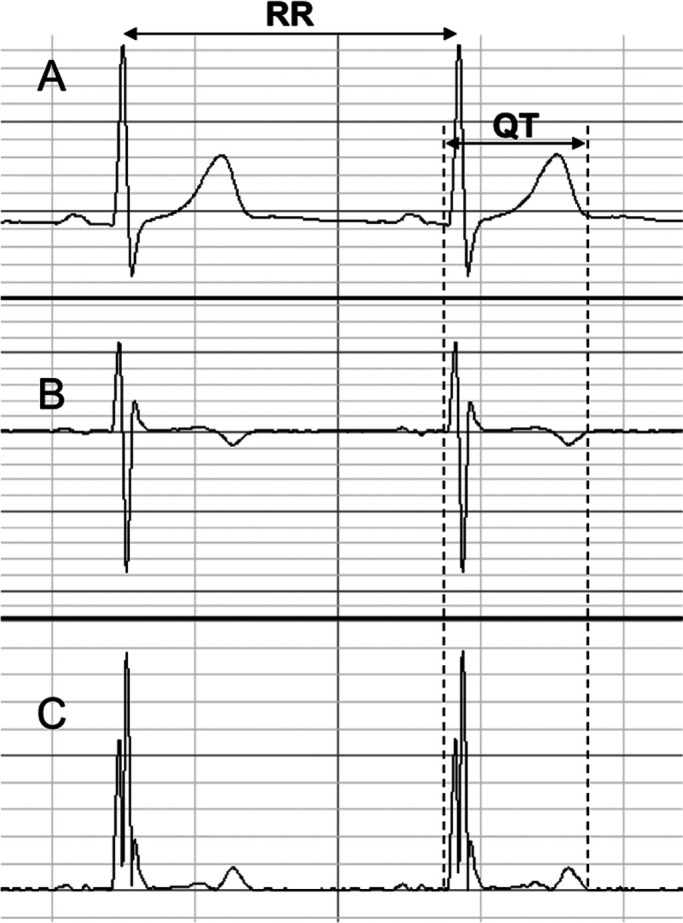
Measurement of RR interval (RR) and QT intervals. Electrocardiograms (ECGs) were recorded by the CM5 lead using a Biopac biological polygraph recording device (Goleta, CA). Q onset, T end, and preceding RR intervals were measured using first derivative processing (B) and absolute functions (C) from 60 consecutive beats with a stable baseline ECG.

We recorded single-lead ECG (CM5 lead) using a Biopac MP-150 bio-polygraph acquisition unit (Biopac Systems Inc., Goleta, CA, USA) as previously reported.^14,15^ Data were recorded from 2 to 3 pm before feeding, with the subject quiet, awake, and placed in the supine position. Automated analysis of the RR interval was performed on ECG recordings of 60 heartbeats with a stable baseline using AcqKnowledge ver. 3.9 analysis software (Biopac Systems Inc.). The endpoint of the T wave was identified using a first-order differentiation processing method, and QT intervals and preceding RR intervals were measured (Figure 1).

### ECG parameters

Standard deviation (SD) of the QT interval (SDQT), SD of the corrected QT interval (SDQTc), SD of the RR interval (SDRR), and root mean square difference of successive RR intervals (rMSSD) were calculated. VR (I–IV) was obtained using the formula proposed by Jensen et al. and Krauss et al. (VR-I: SDQT/SDRR, VR-II: SDQT/rMSSD, VR-III: SDQTc/SDRR, and VR-IV: SDQTc/rMSSD).^12,13^ The Fridericia formula (QTc=QT/3√RR) was used for heart rate correction. Based on the proposal of the American College of Obstetricians and Gynecologists,^16^ preterm infants were defined as neonates born at <37 weeks of gestation.

### Statistical analysis

Data are presented as mean±SD. Comparisons between two groups of data were made with the unpaired Student’s t-test or Mann–Whitney U-test, as appropriate. Frequencies were compared using a chi-squared test. Linear regression analysis was performed by Pearson’s correlation. Further, a multivariable model was constructed to adjust for gestational age, sex, and birth weight. P values of <0.05 were considered statistically significant. The statistical software package JMP (SAS Institute Inc., Cary, NC, USA) was used for analyses.

## Results

### Characteristics of subjects

Twenty-eight preterm infants and 181 full-term (control) infants were included in the study. Birth weight and height, as well as height and weight at 1-month postnatal checkup, were significantly lower in preterm infants compared with full-term infants (Table 1). There were no sex differences in either group.

### Comparison of ECG parameters between the two groups

There were no significant differences in ECG parameters (heart rate, RR, QRS, QT, and QTc) between the two groups (Table 2).

### Relationship between VR (I–IV) and gestational age, birth weight, and Apgar score in all subjects

Associations between VR and gestational age, birth weight, and Apgar score were assessed using linear regression analysis (Table 3). Weak negative correlations were observed between VR-I, -III, and -IV, as well as gestational age. However, there was no significant relationship between birth weight and each VR value, except for a weak negative correlation between VR-IV and birth weight (r=–0.148, p=0.033). No correlation was observed between VR and Apgar score. In a linear multiple regression analysis that included gestational age, sex, and birth weight, only gestational age was significantly associated with VR (I and III) (Table 4).

### Comparison of VR values (I–IV) between the two groups

All VR values (mean±SD) were significantly higher in preterm infants compared with full-term infants (Table 5).

## Discussion

In the present study, we analyzed the ECG parameters of 1-month-old healthy infants. We found that VR was impaired in preterm infants and showed a moderately negative correlation with gestational age, but not birth weight. This suggests that autonomic nervous control of the heart could be dependent on gestational age rather than weight at birth. The VR proposed by Jansen et al.^12^ is a parameter in which the QT interval varies according to the preceding RR interval in the cardiac cycle. They reported that, in patients with old myocardial infarction, arrhythmic events commonly occurred in patients with a high VR.^17^ Krauss et al. modified Jensen et al.’s original VR-I equation by replacing SDRR with rMSSD, which represents vagal nerve activity in the heart and respiratory sinus arrhythmia, in their new equation for VR-II and VR-IV.^13,18^

In this study, heart rate variability (SDRR) was significantly decreased in preterm infants, which might contribute to the increase in VR-I. Similarly, rMSSD, which is an index of respiratory vagal nerve activity that causes fluctuation of RR interval, was decreased in preterm infants, thus contributing the increase in VR-II. Regardless, VR could be understood by the ventricular myocardial electrical instability (SDQT) modulated by autonomic tone. In our study of apparently healthy infants, consistent results were obtained when using VR-I, VR-III/VR-II, or VR-IV, suggesting that heart rate correction might not affect the results. Thus, whether heart rate correction is needed in assessing QT variability or VR remains to be elucidated.

As previously demonstrated in clinical studies, the autonomic nerve balance predominantly elicits sympathetic nerve activity in infants with a low gestational age. Currently, autonomic nervous dysfunction is considered to be one cause of SIDS.^9^ SIDS infants show poor vagal nerve activity or elevated sympathetic nerve activity, a long QT interval, low HRV, and elevated basic heart rate compared with healthy infants.^19,20^ Theoretically, HRV can be used to identify autonomic dysfunction of the sinus node, but cannot detect abnormal ventricular repolarization of the heart, which leads to lethal ventricular arrhythmias. However, VR can be used to detect autonomic nervous dysfunction and repolarization instability of the ventricular myocardium. Furthermore, VR can detect proarrhythmic substrates associated with ion channel-related diseases, such as long QT syndrome and other cardiac repolarization abnormalities. Few studies have reported the VR of infants who have cardiac disease. Interestingly, Eryu et al. demonstrated a positive correlation between VR and left-to-right shunt ratio in patients with atrial septal defects.^21^ They suggested that hemodynamic abnormality may affect SDQT and variance of the QT interval, and noted that VR can be used to determine the extent of latent heart failure. Thus, evaluation of VR will be a useful method for assessment of pediatric disease in the future.

## Limitations

This study has some limitations. First, this was a single-center study with a limited number of patients. Second, all ECG recordings were performed in the supine position with the subjects quiet and awake. Therefore, the results cannot be applied to infants while they are asleep. Third, detailed information that could potentially affect maturation of the autonomic nervous system, such as fetal, placenta/umbilical cord, and maternal factors, was not included in our analysis. Finally, as our study included only apparently healthy infants, it is unclear whether our results could be applied to infants with latent or subclinical myocardial abnormalities such as hereditary channelopathies. As such, this should be explored in the future studies.

## Conclusion

VR was impaired in preterm infants assessed at 1 month after birth, suggesting immaturity of their cardiac autonomic nervous system and ventricular myocardial repolarization.

All authors have read and approved the final version of the manuscript.

## Figures and Tables

**Table1:** Characteristics of subjects for the total sample stratified by gestational age

	<37 weeks	≥37 weeks	p value
Number	28	181	
Male/Female	16/12	109/72	0.683
Gestational Age (weeks)	35.6±1.1	38.8±1.1	<0.0001
Birth weight (g)	2330.3±294.7	3008.2±374.3	<0.0001
Birth Height (cm)	45.4±2.4	48.6±2.0	<0.0001
1-Month Weight	3181.8±474.9	4010.3±544.3	<0.0001
1-Month Height	49.2±2.5	52.6±2.2	<0.0001

Values are expressed as mean±SD. Comparisons between two groups of data were made with the chi-squared test or unpaired Student’s t-test.

**Table2:** Electrocardiogram characteristics stratified by gestational age

	<37 weeks	≥37 weeks	p value
HR (bpm)	167.8±16.5	163.1±18.2	0.197
RR (ms)	361.3±36.7	373.1±43.4	0.172
QRS (ms)	63.7±5.7	64.6±4.5	0.301
QT (ms)	246.1±18.6	250.0±20.0	0.339
QTc (ms)	345.5±15.8	347.3±17.9	0.615

Values are expressed as mean±SD. Comparisons between two groups of data were made with the Student’s t-test. Bpm, beats per minute; HR, heart rate; QRS, Q, R, and S wave complex; QT, QT interval; QTc, QT interval corrected by the Fridericia formula; RR, RR interval.

**Table3:** Correlation between variability ratios and subject characteristics

	VR	r	p
Gestational age	I	–0.203	0.003
	II	–0.134	0.053
	III	–0.220	0.001
	IV	–0.164	0.018
Birth weight	I	–0.097	0.163
	II	–0.129	0.064
	III	–0.106	0.128
	IV	–0.148	0.033
Apgar score (1-min)	I	0.054	0.436
	II	0.042	0.554
	III	0.035	0.620
	IV	0.008	0.909
Apgar score (5-min)	I	0.081	0.244
	II	0.086	0.210
	III	0.073	0.292
	IV	0.068	0.328

P values of <0.05 were considered statistically significant by Pearson’s correlation. VR, variability ratio.

**Table4:** Multiple linear regression analysis

	VR-I	VR-II	VR-III	VR-IV
Gestational age	0.0069	0.2986	0.0038	0.1785
Birth weight	0.4580	0.5173	0.4512	0.4930
Sex	0.5819	0.5242	0.6801	0.6544

Multiple linear regression analysis that included gestational age, sex, and birth weight; only gestational age was significantly associated with VR (I and III). VR, variability ratio.

**Table5:** Comparison of variability ratios stratified by gestational age

	<37 weeks (n=28)	≥37 weeks (n=181)	p value
VR I (mean±SD)	0.84±0.52	0.57±0.34	0.019
VR II (mean±SD)	1.34±0.62	1.08±0.57	0.035
VR III (mean±SD)	1.17±0.76	0.79±0.49	0.022
VR IV (mean±SD)	1.80±0.82	1.46±0.80	0.030
SDQT (mean±SD)	5.37±1.55	5.28±1.56	0.764
SDQTc (mean±SD)	7.23±1.97	7.14±2.08	0.797
SDRR (mean±SD)	8.93±5.73	12.0±6.9	0.004
rMSSD (mean±SD)	4.65±2.21	6.15±3.18	0.005

Values are expressed as mean±SD. Comparisons between two groups of data were made with the Mann–Whitney U-test. rMSSD; root mean square difference of successive RR intervals; SDQT, standard deviation of QT intervals; SDQTc, standard deviation of corrected QT intervals by the Fridericia’s formula; SDRR; standard deviation of RR intervals; VR, variability ratio.
